# Concurrent genome and epigenome editing by CRISPR-mediated sequence replacement

**DOI:** 10.1186/s12915-019-0711-z

**Published:** 2019-11-18

**Authors:** Jes Alexander, Gregory M. Findlay, Martin Kircher, Jay Shendure

**Affiliations:** 1 Department of Genome Sciences, University of Washington, Seattle, WA USA; 2 Department of Radiation Oncology, School of Medicine, University of Washington, Seattle, WA USA; 3 Brotman Baty Institute for Precision Medicine, Seattle, WA USA; 4 Allen Discovery Center for Cell Lineage, Seattle, WA USA; 5 Howard Hughes Medical Institute, University of Washington, Seattle, WA USA

**Keywords:** CRISPR/Cas9, Genome editing, Epigenome editing, DNA methylation, *HPRT1*, Gene silencing

## Abstract

**Background:**

Recent advances in genome editing have facilitated the direct manipulation of not only the genome, but also the epigenome. Genome editing is typically performed by introducing a single CRISPR/Cas9-mediated double-strand break (DSB), followed by non-homologous end joining (NHEJ)- or homology-directed repair-mediated repair. Epigenome editing, and in particular methylation of CpG dinucleotides, can be performed using catalytically inactive Cas9 (dCas9) fused to a methyltransferase domain. However, for investigations of the role of methylation in gene silencing, studies based on dCas9-methyltransferase have limited resolution and are potentially confounded by the effects of binding of the fusion protein. As an alternative strategy for epigenome editing, we tested CRISPR/Cas9 dual cutting of the genome in the presence of in vitro methylated exogenous DNA, with the aim of driving replacement of the DNA sequence intervening the dual cuts via NHEJ.

**Results:**

In a proof of concept at the *HPRT1* promoter, successful replacement events with heavily methylated alleles of a CpG island resulted in functional silencing of the *HPRT1* gene. Although still limited in efficiency, our study demonstrates concurrent epigenome and genome editing in a single event.

**Conclusions:**

This study opens the door to investigations of the functional consequences of methylation patterns at single CpG dinucleotide resolution. Our results furthermore support the conclusion that promoter methylation is sufficient to functionally silence gene expression.

## Background

Mammalian genome editing has become much more straightforward with the discovery of CRISPR systems. Conventional genome editing with CRISPR uses the endonuclease Cas9 to cut the genome at a guide RNA-specified location, which is followed by endogenous DNA repair [1]. The targeting of the Cas9 cut is programmed by a guide RNA which has homology to the sequence that will be cut by Cas9. DNA repair occurs through two main pathways: homology-directed repair (HDR) and non-homologous end joining (NHEJ). HDR-mediated genome editing requires an exogenous DNA repair template bearing homology arms that are used in homologous recombination of the template with the genome, resulting in a precise change at the position of the programmed cut. In contrast, NHEJ-mediated genome editing simply involves religating the broken ends, but this occasionally results in small insertions or deletions, i.e., an imprecise change at the position of the programmed cut. However, if an exogenous DNA template is provided, it can be inserted at the location of the programmed cut by NHEJ-mediated ligation [2]. If dual cuts are programmed nearby to one another, NHEJ-mediated ligation at both double-stranded breaks can result in the replacement of the intervening sequence with an exogenous DNA template [3].

Although the ability to edit the base sequence of the genome is very useful, much of the information that carries cell type-specific properties, such as gene expression, is encoded at an epigenetic level. CpG island methylation is one such layer of epigenetic regulation [4, 5]. CpG dinucleotide methylation is important in both normal development as well as disease, but the mechanisms by which it contributes to the regulation or dysregulation of gene expression remain poorly understood [6, 7].

Editing of DNA methylation has previously been demonstrated by two approaches. In a first approach based on site-specific recombinases such as Cre-*loxP*, *loxP* sites are integrated into the genome at a locus of interest; an in vitro methylated plasmid with *loxP* sites is then transfected and Cre recombinase expressed; this drives recombination of the in vitro methylated DNA into the genome at the locus of interest [8–10]. This approach is highly efficient, but major drawbacks include that the *loxP* sites must be engineered into the genome first, and these sites remain in the genome even after recombination.

A second, recently demonstrated approach uses a catalytically inactive Cas9, as a targeting domain, fused to a DNA methyltransferase domain, for methylation of CpG dinucleotides [11–18]. This approach has lower efficiency and results in methylation of multiple CpGs surrounding the target site, requiring multiple guides if the goal is to methylate a region. In the case of a CpG island, guide design can be complicated by low sequence complexity and targeting ambiguities. For investigations of the functional consequences of methylation, a limitation of this approach is that it fails to discriminate between the consequences of the binding of the fusion protein vs. methylation itself.

**Fig. 1:**
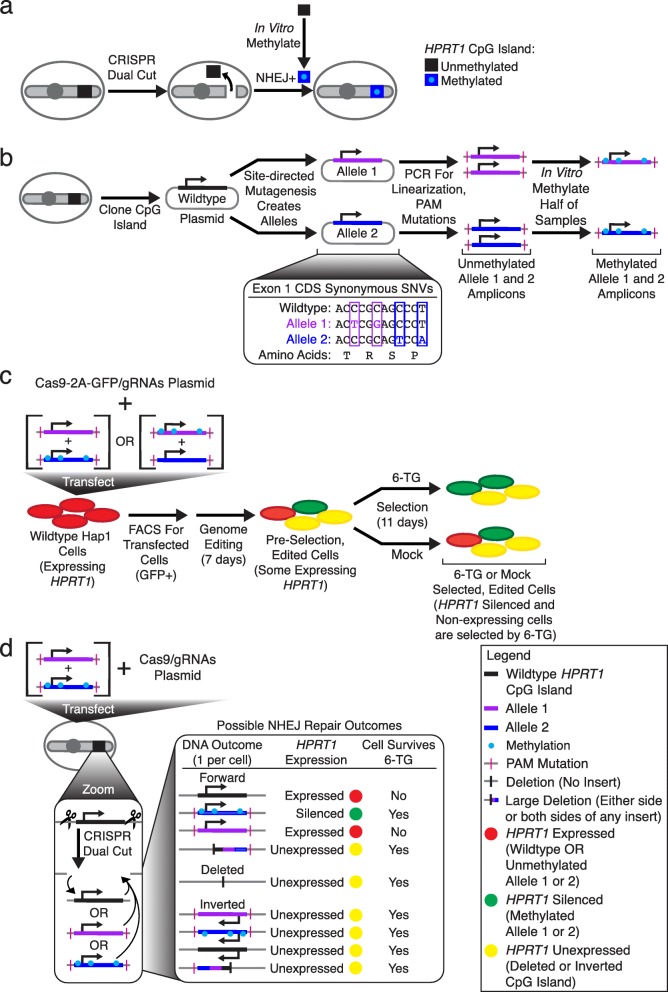
Experimental design. **a** Overview of the experimental approach showing CRISPR dual cuts for removing and replacing the *HPRT1* CpG island with an in vitro methylated DNA sequence through NHEJ-mediated repair. **b** The *HPRT1* CpG island was cloned, and synonymous coding SNVs were introduced to create two distinguishable alleles (blue and purple). Cloned CpG island alleles were PCR amplified for linearization and to incorporate PAM mutations. Portions of the resulting amplicons were in vitro methylated (cyan) with M.SssI. **c** For each replicate, the methylated version of one allele amplicon and the unmethylated version of the other allele amplicon, together with plasmids bearing Cas9-2A-GFP and two gRNAs, were co-transfected into Hap1 cells. In one plate of Hap1 cells, allele 1 was methylated and allele 2 was not, and in a parallel experiment, allele 2 was methylated and allele 1 was not. Transfected cells were sorted by FACS and re-plated for genome editing. Edited cells were then either selected with 6-TG, which will select for cells that do not express *HPRT1*, or mock selected with DMSO. Cells were harvested before and after selection, DNA was extracted, and the relevant regions PCR amplified and sequenced. The alleles allow tracking of the inserted methylated vs. unmethylated CpG island amplicons without requiring bisulfite conversion. The relative frequencies of the methylated and unmethylated alleles were calculated and compared between the 6-TG-selected, mock-selected, and pre-selection cells. **d** Potential outcomes of genome editing are shown for a hypothetical single cell from a single replicate. After a CRISPR dual cut, the possible outcomes at the DNA level are a deletion of the CpG island, re-insertion of the original wild-type CpG island that was cut out, or insertion of the methylated or unmethylated alleles that were transfected in. Inserted CpG islands can be inserted in an inverted or forward orientation. *HPRT1* will be expressed if either the original wild-type or the unmethylated allele is inserted, but will no longer be expressed if a deletion or inversion occurs. Insertion of a forward-oriented, methylated allele should result in methylation-induced silencing. Finally, cells are expected to survive 6-TG selection if they no longer express *HPRT1*, which can be a consequence of methylation-induced silencing, deletion of the CpG island, or inversion of the CpG island. Therefore, upon sequencing after 6-TG selection, if the methylated allele is inserted, we predicted that its relative frequency will be increased as compared to the unmethylated allele.

We wondered whether it would instead be possible to achieve epigenome editing with respect to CpG methylation by using CRISPR/Cas9 to introduce DSBs at two nearby locations, followed by replacement of the intervening segment with a transfected, in vitro methylated version of the same sequence via NHEJ-mediated ligation (Fig. 1a). This strategy has the potential to enable methylation of an entire CpG island (hundreds to thousands of bases) with only two guides. It would also facilitate the introduction of precise, complex patterns of methylation or even of other DNA modifications. Finally, it opens the door to concurrent genome and epigenome editing (i.e., if the exogenous, methylated segment differed in its base sequence from the endogenous segment). To test this approach, we targeted methylation to the CpG island of *HPRT1* in human Hap1 cells [19]. *HPRT1* is a housekeeping gene with the special property that loss of its expression, whether by silencing or mutation, results in resistance to 6-thioguanine (6-TG), a chemotherapeutic purine analog. The Hap1 cell line is haploid, which means that modification of only a single copy of the *HPRT1* locus is required to observe this phenotype.

## Results

We attempted to replace the *HPRT1* CpG island with in vitro methylated DNA using CRISPR-mediated NHEJ (Fig. 1a). To this end, the *HPRT1* CpG island, which overlaps with the first exon of *HPRT1* including a portion of the ORF, was cloned from human genomic DNA (Fig. 1b). Two synonymous SNVs were introduced into the coding sequence of the first exon in the cloned plasmid construct to generate a first allele that was distinguishable from the wild-type CpG island sequence. From the starting construct, a second allele was also created by introducing two synonymous SNVs at different positions than used for the first allele. Since the positions used for the synonymous SNVs in the two alleles were different, the alleles were distinguishable from one another as well as from the wild-type sequence. The CpG island alleles were PCR-amplified to linearize them and then in vitro methylated with the enzyme M.SssI. Through the primers used for this PCR, mutations were introduced to the locations corresponding to the PAM site of the intended guide RNA targets, in order to reduce the probability of re-cutting by Cas9 after any successful insertion events (Fig. 1b; Additional file 1: Figure S1).

Methylated allele 1 and unmethylated allele 2 amplicons, along with plasmids directing expression of Cas9-2A-GFP and guide RNAs targeting the ends of the 1120-bp *HPRT1* CpG island, were co-transfected into a single plate of Hap1 cells. The reciprocal experiment, i.e., using a methylated version of allele 2 and an unmethylated version of allele 1, was performed in parallel, as a form of replication as well as to control for any effects of the synonymous mutations (Fig. 1c). Both the primary and reciprocal experiments were performed in triplicate. A key point is that with this experimental design, the alleles allow one to infer whether the methylated or unmethylated amplicon was inserted, without requiring bisulfite conversion prior to sequencing.

At 48 h after transfection, > 100,000 GFP-positive cells were sorted by FACS and put back into culture for 7 days. GFP positivity indicates that these cells were transfected successfully. At this point, half of the cells from each plate were harvested (“pre-selection” in Fig. 1c) and the remaining half of the cells were split to two dishes. To one dish, 6-TG was added as a selection agent (“6-TG selected” in Fig. 1c), and to the other dish, DMSO was added as a vehicle control (“mock selected” in Fig. 1c). After 11 days, cells were harvested, genomic DNA was extracted, and the *HPRT1* CpG island was PCR amplified and sequenced.

Based on sequencing, the relative frequencies of methylated and unmethylated alleles were calculated and compared between pre-selection, mock-selected, and 6-TG-selected samples. These frequencies are dependent on the outcomes of genome editing, which lead to survival or death under 6-TG selection (Fig. 1d). Possible editing outcomes include deletion of the intervening segment, re-insertion of the original wild-type CpG island, or insertion of the transfected methylated or unmethylated allele. Additionally, the wild-type CpG island or methylated or unmethylated alleles can potentially be inserted in the original forward or an inverted orientation. Since Hap1 cells are haploid, only one of these editing outcomes is expected per cell. Insertion of the methylated allele in the forward orientation might be expected to result in methylation-induced silencing of *HPRT1*, while a deletion or any inversion would result in loss of expression. Cells with silencing or loss of expression of *HPRT1* are expected to survive 6-TG selection, while those with expression are expected to be strongly selected against.

We first sequenced the allele-defining SNVs and the surrounding portion of exon 1 using short-read Illumina sequencing. For this, a nested PCR approach was employed with one outer nest PCR primer upstream of the 5′ cut site and one between the cut sites (Additional file 2: Figure S2). The inner nest amplified a 44-bp region including the allele-defining SNVs in the exon 1 CDS and a small portion of the promoter. The advantage of this nested approach is that it prevented amplification and sequencing of any random integrations at other positions in the genome, as well as on-target inversions or deletions of the intervening segment. Because these other outcomes are excluded, our expectations for this experiment were as follows: If the methylated allele is inserted, 6-TG selection should result in an increase in the frequency of the methylated allele compared to the unmethylated allele (quantifiable by sequencing of the allele-defining SNVs). In contrast, in the pre-selection and mock selection samples, no difference in the frequency of methylated and unmethylated alleles was predicted. On the other hand, a limitation of the nested approach is that we are blind to any NHEJ-mediated indels at the two cut sites themselves. However, in any case with Illumina sequencing, we could not sequence both the allele-defining SNVs and the cut sites in the same read, simply because the reads are too short (in principle, this could be done with paired reads, but the amplicons would be too large for compatibility with Illumina sequencing). We return to this issue and the question of whether there are consequential NHEJ-mediated indels at the individual cut sites further below.

**Fig. 2:**
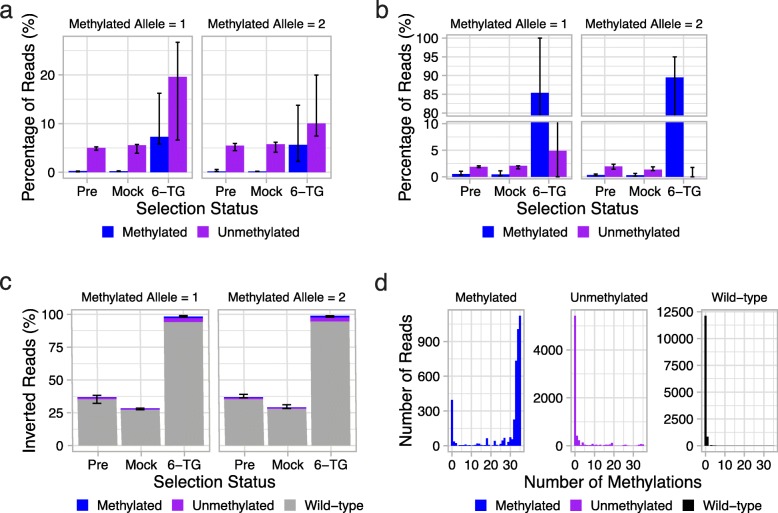
Methylation of the *HPRT1* CpG island by CRISPR-mediated sequence replacement results in *HPRT1* silencing. **a** Percentages of Illumina sequencing reads assigned to methylated and unmethylated inserted alleles by SNVs, grouped by selection status (Pre, pre-selection; Mock, mock selection; 6-TG, 6-TG selection). Although both are enriched, methylated inserted alleles are more enriched than unmethylated inserted alleles after 6-TG selection. Wild-type sequences are not shown, but are included in percentages. The first panel shows the experiment where allele 1 was methylated and allele 2 unmethylated; the second panel shows the reciprocal experiment. Error bars show the range of triplicates. **b** Percentages of PacBio sequencing reads assigned to “exact matching” methylated and unmethylated inserted alleles by SNVs, grouped by selection status (Pre, pre-selection; Mock, mock selection; 6-TG, 6-TG selection). Methylated inserted alleles, but not unmethylated inserted alleles, are strongly enriched by selection. Sequences were only counted if they were in the forward orientation and exactly matched the promoter, exon 1, splice donor, PAM mutation, and one of three sets of allele-defining SNVs (wild-type, allele 1, or allele 2). Wild-type sequences are not shown, but are included in percentages. Error bars show the range of triplicates. Note that the *y*-axis is gapped and contains two scales, to increase resolution in 0–10% range. **c** Percentages of PacBio sequencing reads assigned to reverse/inverted orientation, grouped by selection status. Deletion events, as well as sequences not meeting the “exact matching” criteria defined above, were not counted. Forward-oriented sequences are not shown, but are included in percentages. The clear pattern is that inverted sequences predominate after 6-TG selection. **d** Observed number of methylated sites upon bisulfite sequencing of methylated, unmethylated, or wild-type alleles of the CpG island, summed across selection conditions. The region contains 35 CpG dinucleotides. Reads are assigned to in vitro methylated or unmethylated alleles, or to unedited wild-type sequence based on synonymous SNVs. In vitro methylated alleles remain heavily methylated, while unmethylated alleles and unedited sequences remain predominantly unmethylated

We quantified the frequencies of the inserted methylated and unmethylated alleles, both pre-selection as well as after 6-TG and mock selection (Fig. 2a). These frequencies were calculated using only counts of forward-oriented methylated, unmethylated, and wild-type alleles, and as noted above, we are blind to any mutations at the cut sites for all of these classes, including the wild-type allele. A first observation is that even pre-selection, the proportion of methylated alleles that are inserted is very low (mean 0.24%). In contrast, the proportion of unmethylated alleles inserted is modest but consistent (mean 5.1%). This suggests that NHEJ-mediated insertion of methylated alleles is markedly less efficient than that of unmethylated alleles. For both methylated and unmethylated alleles, the proportions after mock selection were largely unchanged. Surprisingly, the effect of 6-TG selection was to increase the percentage of *both* the inserted methylated and unmethylated alleles, relative to the wild-type allele. However, the fold change for 6-TG selection over mock selection of the methylated allele was much greater than that of the unmethylated allele, suggesting an enrichment for the methylated allele, which is consistent with methylation-induced silencing of *HPRT1* (mean fold change for methylated vs. unmethylated, 41.0 vs. 3.0; log-transformed, paired *t*-test *p* ≈ 0.002).

Given that the above experiments were blind to the cut sites, we speculated that the unexpected increase in inserted, unmethylated alleles upon selection (Fig. 2a) might have resulted from loss of expression due to repair-induced indels at the end(s) of the of the CpG island insert (in the first intron or 5′ UTR; as noted above, we were not able to observe these junctions in the experiment represented in Fig. 2a), or alternatively from mutations in the promoter, exon 1 coding sequence, or splice donor in the CpG island insert introduced by PCR. To test this, we amplified a ~ 2-kb region including the entire CpG island, with primers positioned ~ 700 bp upstream of one cut site and ~ 165 bp downstream of the other cut site (Additional file 3: Figure S3). We sequenced these amplicons using Pacific Biosciences (PacBio) instruments (the “Materials and methods” section).

Circular consensus sequence (CCS) calling was performed to a mean CCS accuracy of 99.4%. In contrast with our Illumina-based sequencing, this approach is expected to recover not only forward-oriented alleles, but also inversions, deletions, and multiple insertions of the intervening sequence. However, we did not attempt to quantify wholesale deletions or multiple insertions of the CpG island for the following reasons. First, we performed a gel extraction step after PCR that removed most deletion events. Second, although the PCR cycling conditions were designed to be able to amplify multiple insertion events, bands representing such longer sequences were not visible on agarose or polyacrylamide gels. Third, even to the extent that either wholesale deletions or multiple insertions are recovered, due to biases in PCR amplification and sequencing towards shorter sequences, it would be very difficult to interpret the counts of sequences of different sizes.

For our first analysis of these PacBio data, sequences were only counted if they were in the forward orientation and moreover exactly matched the promoter, exon 1, splice donor, expected PAM site for a given allele, and one of three sets of allele-defining SNVs (wild-type, allele 1, or allele 2), i.e., excluding inversions as well as sequences containing PCR errors or repair-induced indels. Because we required observation of the expected PAM sites for a given allele, indels at either cut site that extend more than 5 bp into the CpG island were excluded from this analysis. In contrast with the Illumina-based results presented in Fig. 2a, after 6-TG selection, we observed markedly higher proportions of methylated inserted alleles than unmethylated inserted alleles (mean 82.8% vs. 8.1%; arcsine square root transformed, paired *t*-test *p* ≈ 0.005) (Fig. 2b; Additional file 4: Table S1). However, as illustrated by the pre-selection and mock selection experiments, the proportion of inserted methylated and unmethylated alleles remained very low in the absence of 6-TG.

We also examined other sequences in the PacBio data, i.e., sequences other than those exactly aligning to forward-oriented wild-type or forward-oriented inserted alleles. For example, one prediction is that 6-TG should also select for alleles inserted in the inverted orientation, regardless of whether it is the wild-type sequence or one of the exogenous inserts. To investigate this, we tabulated sequences that exactly matched the promoter, exon 1, splice donor, PAM mutation, and any of the three sets of allele-defining SNVs (wild-type, allele, 1 or allele 2), in *either* orientation. Events involving wholesale deletion of the CpG island were again excluded. Collapsing all alleles in each orientation, we observe that the proportion of forward-oriented alleles was modestly higher in both the pre-selection and mock selection samples (mean 63.4% and 71.1% forward oriented, respectively). Although, percentages closer to 50/50 might have been expected, the deviation towards forward-oriented alleles is likely because the calculation includes wild-type alleles that were not fully cut out (e.g., either because of incomplete editing or NHEJ-mediated indels at one of the cut sites). However, after 6-TG selection, the overwhelming majority of sequences were in the reverse/inverted orientation (mean 98.6% reverse oriented) (Fig. 2c; Additional file 4: Table S1). This confirms that 6-TG selection was nearly complete, particularly as the forward-oriented sequences observed after 6-TG selection were dominated by the methylated, inserted alleles (Fig. 2b).

Although we observe that the forward-oriented, methylated allele is strongly selected for by 6-TG, we sought to confirm that its in vitro methylation is maintained after transfection and insertion, and thus might plausibly cause silencing of *HPRT1* and the consequent strong selection. We therefore performed bisulfite sequencing on a region of the CpG island including the allele-defining SNVs and 35 surrounding CpGs (Additional file 5: Figure S4). We observe that the in vitro methylated allele remained heavily methylated in the pre-selection, mock selection, and 6-TG selection samples, whereas the unmethylated allele and the wild-type sequence remained predominantly unmethylated in all samples (Fig. 2d). Of note, bisulfite sequencing of this same region in untransfected Hap1 cells harvested after mock selection exhibited a lack of methylation similar to the wild-type sequences of the transfected cells (data not shown). Consistent with this, 6-TG selection of untransfected Hap1 cells killed all cells, confirming that the *HPRT1* gene was not silenced by methylation without our intervention.

Estimates of the rate of insertion of the methylated allele based on data in Fig. 2b are not based on all sequences and are therefore not exact. In our view, it is not possible to obtain a precise insertion rate from these data because of size biases in PCR amplification and sequencing, which vastly overestimate the number of the shorter deletion sequences. However, in an attempt to get a better estimate, we recalculated insertion rates, but this time including all sequences, except wholesale deletions of the intervening sequence, that could be aligned to the CpG island in either the forward or the inverted orientation in the total count, i.e., the denominator. Sequences were included in this total count regardless of whether or not they could be assigned to either the allele or the wild-type sequence, and indels larger than 5 bases were also included (in the previous calculations, sequences with indels larger than 5 bases were effectively filtered out because of the requirement that the PAM sites, which are 6 bases from the cut sites, match). Using only sequences that could be assigned to the methylated allele with a perfect match in the promoter, exon 1, splice donor, and PAM mutations and allowing up to 5 bp indels on either side, the methylated allele represented 0.72% of reads. If no indels were allowed, 0.12% of reads were the methylated allele. When the pre-selection and mock selection samples were combined and averaged for an estimate of the insertion rate without selection and up to 5 bp indels were allowed, the methylated allele represented 0.16% of reads. If no indels were allowed, the methylated allele represented 0.03% of reads.

Although our strategy remains challenged by the much higher rates of deletion or inversion over insertion of the methylated inserts, our observations nonetheless support the conclusions that (a) we successfully used CRISPR/NHEJ to replace the *HPRT1* CpG island with an in vitro methylated allele; (b) this methylation was maintained after insertion to the genome, at least over the course of our 11-day experiment; and (c) this methylation was sufficient to functionally silence the *HPRT1* gene.

Why are unmethylated alleles frequent upon 6-TG selection in the Illumina-based results but not the PacBio-based results, given that this is the same experiment? As the main difference between these analyses involves the former analysis being blind to the larger region vs. the latter including but only allowing small indels at the repair junctions, we speculated that large repair-induced indels (included in the Illumina-based analysis of Fig. 2a, but analytically excluded from the PacBio-based analysis of Fig. 2b) may result in a subset of forward-oriented, unmethylated inserts being positively selected.

**Fig. 3:**
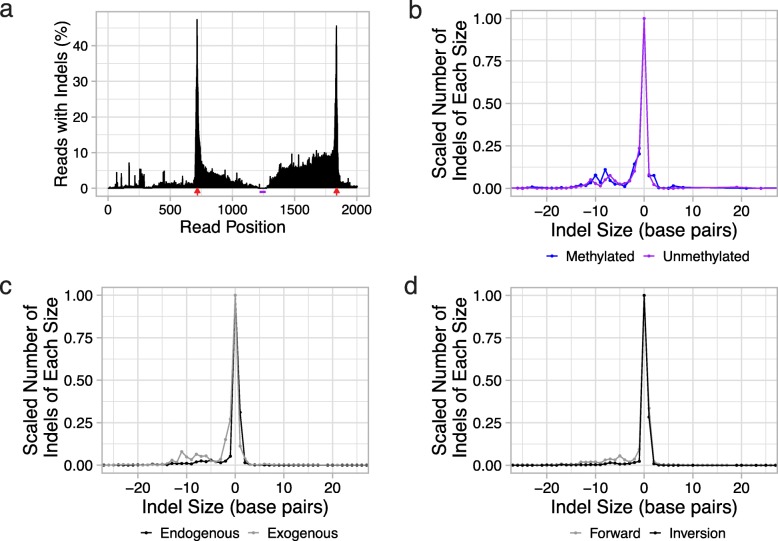
The positional and size distribution of indels, in relation to methylation status, insertion type, and orientation. **a** Percentage of reads with an indel at positions along the PacBio sequenced region. The same subset of reads used in Fig. 2a is included here (6-TG selected, both methylated and unmethylated, perfect match on the allele-defining SNVs and the surrounding portion of exon 1). Red arrowheads indicate the CRISPR/Cas9 cut sites. The purple bar marks the region of exon 1 surrounding the allele-defining SNVs. The distribution of indels is highest at the CRISPR/Cas9 cut sites, but many reads have indels within the CpG island as well. **b** Indel distributions at repair junctions of methylated (blue) or unmethylated (purple) alleles. **c** Indel distributions at repair junctions from events involving exogenous inserts (gray) or endogenous inserts (forward-oriented and inverted wild-type sequences; black). **d** Indel distributions at repair junctions from forward-oriented wild-type sequences (gray) or inverted wild-type sequences (black). The numbers of indels (*y*-axis) were scaled so that the maximum number for any indel size (*x*-axis) for a given distribution was one to allow easier comparison between distributions. Negative numbers for indel size represent deletions, positive numbers represent insertions, and sequences without any repair junction indels have an indel size of zero

To assess this and related questions, we further analyzed the PacBio sequencing data to explore the indel patterns at the cut sites. First, we asked why, in the Illumina short-read sequencing, 6-TG selection resulted in enrichment of both methylated and unmethylated alleles rather than just methylated alleles (Fig. 2a, b). As discussed above, comparison of the Illumina short-read sequencing and PacBio sequencing data suggested that larger indels impacting functional regions of the CpG island insert, i.e., the 5′ UTR, promoter, exon 1, or splice donor sequences, might cause loss of expression of *HPRT1*, resulting in selection of these indel-bearing, unmethylated sequences by 6-TG. We formally addressed the question by analyzing the distribution of indels across the region subjected to PacBio sequencing (Fig. 3a). To facilitate comparison, inclusion criteria were identical to those used for analysis of Illumina reads (both methylated and unmethylated allele sequences selected by 6-TG, with a perfect match of the allele-defining SNVs and the surrounding region of exon 1). As expected, the distribution of indel sites had peaks at both CRISPR/Cas9 cut sites (Fig. 3a). Notably, many indels extended from the flanking CRISPR/Cas9 cut sites into the interior of the CpG island encompassing functional regions involved in *HPRT1* expression. Such indels are predicted to result in loss of expression of *HPRT1*. Since these regions were not visible to Illumina short-read sequencing, indel-containing alleles were included in the results shown in Fig. 2a, but were excluded by our sequencing matching requirements with PacBio for the results shown in Fig. 2b. Overall, we conclude that any modest enrichment of unmethylated alleles after 6-TG selection was likely due to these alleles containing indels extending into functional regions of the CpG island (Additional file 6: Figure S5).

Next, we examined the potential effects of methylation on indel patterns in CRISPR/NHEJ-mediated sequence replacement. We started by asking whether there are differences in the rates of insertion of methylated vs. unmethylated alleles. A caveat of this analysis is that it is unclear if the 100,000 cells transfected are sufficient to accurately quantify the frequency of insertion events, which were rare (Additional file 4: Table S1). Nonetheless, combining alleles and observations in both orientations, we found that the unmethylated allele was consistently inserted more frequently than the methylated allele (0.65% methylated vs. 2.37% unmethylated in pre-selection; 0.60% methylated vs. 2.06% unmethylated in mock selection). These differences were consistent between the forward and reverse orientations. There are reports that some double-strand breaks are repaired differently in methylated than unmethylated DNA; it is possible that such differences may also affect the relative rates of insertion of methylated vs. unmethylated fragments [20, 21].

If these methylated vs. unmethylated inserts are handled differently, it might, but not necessarily, be reflected in a difference in the rates of repair-associated indels. We therefore examined the rates of indels at the flanking CRISPR/Cas9 cut sites, excluding sequences from 6-TG-selected samples. We did not find a rate difference between the methylated and unmethylated alleles (48.9% vs. 50.9%, Fisher’s exact test *p* ≈ 0.3) and furthermore observed similar distributions of indel sizes for methylated vs. unmethylated sequences (Fig. 3b).

However, we did observe a higher rate of indels for exogenous inserts (i.e., either methylated or unmethylated alleles in either orientation) as compared with endogenous inserts (50.4% vs. 40.6%, Fisher’s exact test *p* < 2.2 × 10^− 16^; size distribution of events in Fig. 3c; counts for endogenous inserts include both forward-oriented and inverted wild-type sequences; of note, whereas all inverted alleles were obviously cut out then reinserted, we cannot distinguish whether forward-oriented sequences were cut out and then re-inserted vs. not). These data suggest that exogenous DNA might be more likely to be inserted if there is exonuclease chew-back during the repair. This result is further supported by the indel distribution of 6-TG-selected methylated and unmethylated alleles, which showed many indels extending from the CRISPR/Cas9 cut sites into the interior of the CpG island (Fig. 3a). We note that three phosphorothioate linkages were incorporated during PCR at both ends of the insert amplicons, because these linkages are supposed to prevent exonuclease chew-back [3]. It is unclear how effective these linkages were, and it is possible that the association between insertion and exonuclease chew-back is simply an artifact of these linkages.

Again excluding 6-TG-selected sequences, we also observed higher rates of indels for forward-oriented wild-type alleles as compared to inverted wild-type alleles (46.8% vs. 27.5%, Fisher’s exact test *p* < 2.2 × 10^− 16^; size distribution of events in Fig. 3d). However, this could simply be due to an increased propensity for indels when the break-repair recreates the wild-type sequence without mutation, because this site becomes a substrate for CRISPR/Cas9 cleavage again. This break-repair cycle can repeat until Cas9 is no longer active or a mutation occurs, explaining the higher rate of observed indels with forward-oriented wild-type alleles.

## Discussion

In this proof-of-concept study, we demonstrate concurrent epigenome and genome editing using CRISPR/Cas9. Our approach was to swap out endogenous DNA for exogenous DNA that was in vitro methylated and furthermore harbored programmed sequence differences. Specifically, we excised the endogenous *HPRT1* CpG island DNA using dual, flanking CRISPR/Cas9 cuts in the presence of transfected, in vitro methylated, SNV containing, exogenous *HPRT1* CpG island DNA. Our results demonstrate that it is possible to directly introduce in vitro methylated DNA into the genome using the NHEJ repair machinery in a targeted manner, and critically, that methylation of the exogenous fragment is maintained and can lead to robust gene silencing.

For targeted methylation, this CRISPR/NHEJ approach represents an alternative to the previously demonstrated dCas9-methyltransferase domain fusion protein approach [11–17]. While both approaches can produce targeted, scarless methylation of genomic DNA, the CRISPR/NHEJ approach is distinguished by the potential to program precisely which subsets of CpG dinucleotides are methylated, e.g., if exogenous inserts with specific patterns of CpG methylation are synthesized. In principle, this CRISPR/NHEJ strategy could be used to investigate the functional consequences of methylation patterns at single-site resolution, e.g., whether specific CpGs or combinations of CpGs are more important than others, and also whether/how these functional consequences depend on local sequence variation. Furthermore, other base modifications, e.g., hydroxymethylation or even non-standard bases, could be introduced into the genome by our approach, perhaps to study how they would be repaired or themselves further modified over subsequent cycles of DNA replication.

At least to our knowledge, this level of resolution is not possible with the dCas9-methyltransferase approach, which non-uniformly methylates sites over a window that may include tens to hundreds of CpGs in a probabilistic manner that depends on proximity to the enzyme [11–17]. Beyond resolution, a further advantage of the CRISPR/NHEJ approach is that it separates the effect of the methylated base from the act of methylation, i.e., functional effects observed with the dCas9-methyltransferase approach may be due to the effects of the fusion protein binding to the CpG island or promoter, rather than the methylated CpGs themselves.

These advantages notwithstanding, there are important practical limitations of our approach. There were three key elements of the experimental design that made this approach successful on the CpG island of *HPRT1*. First, rather than using RNA sequencing as a readout, we used selection for gene silencing and PacBio long-read DNA sequencing as a functional readout. This was necessary because of the diversity of editing outcomes and the fact that the vast majority did not involve the methylated allele (Fig. 2a; Fig. 3a). Second, since selection was required, we chose to target methylation to the *HPRT1* CpG Island. Expression of this gene in the presence of a small molecule chemotherapeutic, 6-TG, results in cell death. This allowed us to enrich for cells in which *HPRT1* had been successfully silenced. Third, we performed our experiments in the Hap1 cell line because it is haploid, such that the phenotype caused by successful insertion of the methylated allele would not be obscured by an unedited, expressed second copy of *HPRT1*, as would be the case with a diploid cell line.

In other experiments, we attempted to apply the CRISPR/NHEJ approach to methylate the CpG island of other genes. However, this proved difficult because of the requirement for a selection-based readout. Towards making such a readout possible on other genes beyond *HPRT1*, we engineered derivative Hap1 cell lines in which target genes were tagged with a negative selection marker such that expression of the gene would result in sensitivity to a small molecule drug, replicating the interaction between 6-TG and the *HPRT1* gene. Unfortunately, we were unable to successfully complete these experiments because of the poor transfection efficiency of the engineered cell lines. Freshly thawed, low passage HAP1 cells have a transfection efficiency of < 5%, and after the many passages required for engineering, this reduced to approximately 0.1%. This low transfection efficiency is compounded by the low rate of NHEJ repair in Hap1 cells. Future studies employing this approach of tagging other genes with negative selection markers will need to use much larger numbers of Hap1 cells or alternative cell lines with similar properties to Hap1 cells, but with better transfection efficiencies.

Finally, an important limitation of our approach, at least in its current form, is the effectively low efficiency of introducing methylation. This study showed much lower methylation rates (< 1%) as compared to the dCas9-methyltransferase fusion protein approach (30–70%) [11–17]. Part of this low efficiency may be due to the specific way in which the experiment was controlled. Since both methylated and unmethylated amplicons were co-transfected, each cell that was successfully transfected was likely to receive many copies of both types of amplicon, causing competition for insertion. Transfecting only methylated amplicons could increase efficiency, but probably only modestly. The low efficiency is likely to be primarily consequent to other factors, including the low transfection efficiency and rate of NHEJ of the Hap1 cell line, the lower integration rate of methylated DNA, and the availability of alternative outcomes that are also selected for, e.g., most prominently reinsertion of the endogenous DNA fragment in an inverted orientation. These limitations are potentially addressable through further modifications of the approach, e.g., optimization of guide RNAs for efficiency of cutting and/or to modulate the distribution of repair outcomes [22].

## Conclusions

In conclusion, in this proof-of-concept study, we demonstrated concurrent epigenome and genome editing of the *HPRT1* CpG island in a single event using dual CRISPR/Cas9 cuts. The direct replacement of the native *HPRT1* CpG island sequence with the methylated exogenous *HPRT1* CpG island sequence resulted in functional *HPRT1* gene silencing. Although challenges remain particularly with respect to efficiency, this approach constitutes a highly programmable new method for studying the direct effects of methylated DNA sequences in their endogenous contexts that may prove broadly useful for understanding the interplay between DNA modifications and gene expression at high resolution.

## Materials and methods

### Generation of HPRT1 CpG island alleles and guide RNAs

The HPRT1 CpG island region (GRCh37/hg19, chrX:133593694-133595157; Additional file 7: Figure S6) was amplified from HeLa S3 DNA using Kapa Hifi Hotstart Readymix (Kapa Biosciences) and primers 1 and 2. Sequences of all primer and oligonucleotides used are in Additional file 8: Table S2. This amplicon was cloned using ClonTech In-Fusion Cloning kit into the pUC19 vector supplied with the kit. Synonymous SNVs were introduced into the cloned *HPRT1* CpG island plasmid by PCR amplification of the entire plasmid with primers 3–6 using Kapa Hifi Hotstart Readymix (Kapa Biosciences) followed by re-circularization of the plasmid using ClonTech In-Fusion Cloning Kit. The synonymous SNVs were placed within the exon 1 coding sequence at genome positions, chrX:133594350 (C to T; allele 1), chrX:133594353 (C to G; allele 2), chrX:133594356 (C to T; allele 2), and chrX:133594359 (T to A; allele 1). For gRNAs, oligonucleotides 7–10 were synthesized by IDT, annealed, and cloned into pX458 plasmid (Addgene plasmid #48138) using ClonTech In-Fusion Cloning kit. The spacer sequences for these gRNAs were from chrX:133593802-133593821 and chrX:133594936-133594955. All cloned sequences were verified by Sanger Sequencing. DNA was extracted for all constructs using Qiagen mini-prep kits following the manufacturer’s instructions on multiple 5 mL cultures.

To generate NHEJ template DNA, cloned alleles were amplified using Kapa Hifi Hotstart Readymix (Kapa Biosciences) and primers 11 and 12 resulting in an amplicon with the same sequence as chrX:133593819-133594938. This sequence is the region expected to be cut out of the genome by the gRNAs cloned above. The primers contain three phosphorothioate linkages at the 5′ end and mutations to destroy protospacer adjacent motif (PAM) sites at genome positions, chrX:133593824 (G to C) and chrX133594933 (C to G). PCR purification was performed using PCR Purification kit (Qiagen). DNA was methylated in vitro using M.SssI methyltransferase (NEB) following the manufacturer’s instructions. To confirm methylation, DNA was digested using the methylation sensitive restriction enzyme, SmaI (NEB), following the manufacturer’s instructions and visualized by polyacrylamide gel (SeaKem LE Agarose, Lonza) and SYBR Gold (Invitrogen). Methylated DNA was cleaned up using a Qiagen PCR Purification kit. All concentrations were determined by using Qubit dsDNA BR kit (Invitrogen).

### Cell culture, transfections, FACS, and selection

The haploid cell line Hap1 was maintained at 37 °C in Iscove’s modified Dulbecco’s medium (ThermoFisher Scientific) supplemented with 10% fetal bovine serum and penicillin/streptomycin. For transfections, cells were treated with 0.05% Trypsin-EDTA (ThermoFisher Scientifc) and reseeded in 10 cm dishes to achieve approximately 50% confluency by the next day. The next day, each plate of cells was transfected with a mixture of both gRNA plasmids and both allele amplicons in a 0.45:0.45:0.05:0.05 ratio with a total of 18 μg of DNA per plate using Turbofectin 8.0 (Origene) and otherwise following the manufacturer’s instructions. For three plates, the allele 1 template was methylated and the allele 2 template was unmethylated. For the other three plates, the allele 2 template was methylated and allele 1 template was unmethylated. Forty-eight hours after transfection, cells were dissociated from plates with trypsin and incubated for 45 min at 37 °C in medium containing 10 μg/mL Hoechst 33342 (ThermoFisher Scientific), a live-cell DNA dye. Fluorescence-activated cell sorting (FACS) was used to retrieve more than 100,000 cells from each plate that were both GFP positive (i.e., transfected) and in the G1 cell cycle phase (i.e., haploid). Sorted cells were placed back into culture in 6-well dishes for 1 week in supplemented medium with media changes every 3 days. At 1 week, each dish of cells was treated with trypsin to dissociate the cells and washed with Dulbecco’s phosphate-buffered saline (ThermoFisher Scientific). Fifty percent of each sample of cells was snap frozen for later DNA extraction, and the other 50 % was split to two wells of a 6-well dish. One of these wells received 5 μM 6-TG (Sigma) in DMSO for negative selection, and the other received DMSO as a control (mock selection). A control plate of untransfected cells was also treated with 5 μM 6-TG to monitor selection status. Cells were cultured for 11 days with media changes and replacement of selection agents every 3 days. At 11 days, cells were treated with trypsin and snap frozen for later DNA extraction.

### DNA extraction and sequencing

DNA and RNA were extracted using a Qiagen Allprep kit as per the manufacturer’s instructions. For Illumina sequencing, a three-round nested PCR with Kapa Hifi Hotstart Readymix and 250 ng of DNA (~ 100,000 genome equivalents) per sample was used to prepare amplicons. The first round of PCR with 3 cycles (primers 13 and 14) added a unique molecular index (UMI), the second round (primers 15 and 16) was for amplification, and the third round (primers 17–27) added flow-cell adapters starting with 1/50 of the second-round reaction as input. Round 2 and 3 PCRs were followed in real time using SYBR Green (Invitrogen) and stopped prior to plateauing. Agencourt Ampure XP bead (Beckman-Coulter) clean-up (1.0×) was performed after each round of PCR. Amplicon DNA from each sample was pooled at equal concentration and sequenced on an Illumina MiSeq using a 2 × 75 cycle paired-end kit with custom sequencing (primers 51 and 52) and index primers (primer 53), but otherwise as per the manufacturer’s instructions.

For Pacific Biosciences sequencing, a two-round nested PCR with Kapa Hifi Hotstart Readymix and 250 ng of DNA per sample was used to prepare amplicons. The first round with 3 cycles added a UMI to some of the samples (primers 28 and 29) or added a UMI and sample barcode to the remaining samples (primers 29 and 32–45), and the second round (primers 30 and 31) was for amplification. To increase the amount of DNA prior to gel extraction for samples without barcodes, a third round of PCR starting with 1/50 of the second-round reaction as input and using the second-round primers was performed. Round 2 and 3 PCRs were followed in real time using SYBR Green (Invitrogen) and stopped prior to plateauing. Using SYBR Gold and blue light for visualization, gel extractions of the approximately 2000 bp band were performed to reduce the number of deletions (approximately 1000 bp) sequenced. For samples without barcodes, different 1.5% agarose gels were used for each sample. For barcoded samples, groups of samples were pooled prior to loading the gel and groups of pools were gel extracted together. A Qiagen Gel Extraction Kit was used as per the manufacturer’s instructions. For samples without barcodes, 500 ng of DNA per sample was used as input into Pacific Biosciences SMRT Bell Template Prep Kit 1.0 for preparation for sequencing as per the manufacturer’s instructions. For samples with barcodes, the gel-extracted DNA pools were mixed at equal concentrations and then prepared for sequencing by the University of Washington PacBio Sequencing Service (UWPBSS). For samples without barcodes, sequencing was performed on an RSII using P6-C4 chemistry by the UWPBSS using one SMRT cell per sample. For samples with barcodes, the library was sequenced on a Sequel SMRT Cell 1 M v3.0.

For bisulfite sequencing, between 420 ng and 1344 ng of DNA per sample was bisulfite converted using Promega MethylEdge Bisulfite Converion kit as per the manufacturer’s instructions. A three-round nested PCR with Kapa Hifi Uracil+ (first and second rounds) and Kapa Hifi Hotstart Readymix (third round) and half of the bisulfite-converted DNA was used to prepare amplicons for Illumina sequencing. The first round was 3 cycles (primers 46 and 47) for adding UMIs, the second round (primers 48 and 49) was for amplification, and third round (primers 17–24 and 50) was for adding flow-cell adapters starting with 1/50 of the second-round reaction as input. Round 2 and 3 PCRs were followed in real time and stopped prior to plateauing. Agencourt Ampure XP bead clean-ups (0.8×) were performed twice after each round of PCR. Amplicon DNA from each sample was pooled and sequenced on a MiSeq using 2 × 250 cycle paired-end kit with custom sequencing and index primers (primers 51–53).

### Sequencing data analysis

For Illumina DNA sequencing, after bcl2fastq (version 2.18, Illumina) was run for demultiplexing, read 2 FASTQ files were converted to FASTA format. Sequences were then converted to their reverse complement and aligned to the HPRT1 CpG island reference (chrX:133594298-133594522) sequence using needleall (version EMBOSS:6.5.7.0, http://emboss.sourceforge.net/apps/release/6.5/emboss/apps/needleall.html). Based on this alignment, sequences were assigned to alleles (allele 1 vs. allele 2 vs. wild type) using allele-defining SNVs. Perfect matches of all bases in a portion of exon 1 (chrX:133594320-133594363) including the coding sequence and at the four SNV positions were required for assignment to an allele group.

For bisulfite sequencing, after bcl2fastq was run for demultiplexing, paired-end reads were merged with PEAR (Paired-End reAd mergeR, version 0.9.6) and discordant pairs were removed [23]. Sequences were then converted to their reverse complement and aligned using needleall to the HPRT1 CpG island reference (chrX:133594321-133594556) sequences consisting of a bisulfite-converted sequence, a bisulfite-converted sequence assuming all CpGs were methylated, and an unconverted sequence. Unique molecular identifiers (UMIs) and HPRT1 CpG island sequences were extracted from the BAM files for each read based on the alignment. The sequences were clustered by UMI, and a consensus sequence was generated for each cluster by simple majority at each position in the sequence. The consensus sequences were then realigned with the reference sequences using needleall. Based on this alignment, sequences were assigned to alleles (allele 1 vs. allele 2 vs. wild type) using the allele-defining SNVs. Perfect matches of all bases in a portion of exon 1, including the coding sequence (chrX:133594296-133594578), and at the 4 SNV positions were required for assignment to an allele group.

For Pacific Biosciences sequencing data, bax2bam (version 0.0.2, Pacific Biosciences, Inc.) was run on the .h5 files for conversion to BAM files. This was followed by circular consensus calling using CCS (version 2.0.0, Pacific Biosciences, Inc.). Sequences from the generated BAM files were converted to their reverse complement, and both the forward and reverse complement sequences were saved to FASTA format. All sequences were aligned using needleall against the reference forward and inverted sequences of the HPRT1 CpG island. Reference sequences included the HPRT1 CpG island sequence and flanking primer sequences to allow UMIs to be captured. Barcodes were also included in the reference sequences for the Sequel SMRT cell sequencing data to assign each read to a sample. The inverted reference was created by inverting the sequence between the CRISPR cut sites, but keeping the flanking sequence unaltered. The UMIs and HPRT1 CpG island sequences were extracted from the BAM alignment files for each read based on alignment coordinates. Again, sequences were clustered by UMI, a consensus sequence calculated and realigned using needleall. Based on this new alignment, sequences were grouped by alleles (allele 1 vs. allele 2 vs. wild type vs. deletion) and orientation (forward vs. inverted) using the four allele-defining SNVs and two PAM mutations. Perfect matches in the promoter, exon 1, and splice donor sequence (chrX:133594124-133594373), and at the allele-defining SNVs and PAM positions were required for assignment to an allele group.

Counts of reads assigned to allele groups were used for Fig. 2, as described in the figure caption. For Fig. 3, indels were counted in reads assigned to allele groups. Specifically for Fig. 3b-d, indels within 5 bp on either side of the expected CRISPR/Cas9 cut sites based on the read alignments above were included in the count. Sizes of these indels were also determined based on the alignment. The deletions could only extend five bases into the insert sequence because the PAM mutations, which were at the sixth base, were required for assignment to an allele group. Unless otherwise noted, custom scripts were written for these analyses using bash, Python, and R programming languages.

## Supplementary information

**Additional file 1: Figure S1.** PCR for generation of CpG Island Allele Amplicon DNA with PAM mutations. Cloned *HPRT1* CpG island plasmid DNA was used as a template for PCR amplification. PAM sites corresponding to the guide RNA target sites are at the ends within the CpG island amplicon. Primer sequences included a mismatch near the 5′ end resulting in incorporation of a mutation in the PAM sequences at the ends of the CpG island allele amplicons.

**Additional file 2: Figure S2.** Nested PCR from Pre-selection, Mock Selected, or 6-TG Selected Cell Genomic DNA for Illumina Sequencing. Genomic DNA was the template for the first round of PCR. In this round, one primer was outside the CRISPR cut sites in the genome, while the other primer was within the cut sites in the *HPRT1* CpG island. The product of the PCR was used as the template for the second round PCR. The second round PCR amplified a 44-bp region including the allele-defining SNVs.

**Additional file 3: Figure S3.** PCR from Pre-selection, Mock Selected, or 6-TG Selected Cell Genomic DNA for Pacific Biosciences Sequencing. Genomic DNA was the template for the first round of PCR. In this round, both primers were outside the CRISPR cut sites in the genome. A unique molecular index (UMI) and a primer binding site were added in this round of PCR. The product of this PCR was used as the template for the second round PCR.

**Additional file 4: Table S1.** Counts of reads including both inverted and forward-oriented sequences that exactly matched the promoter, exon 1, splice donor, PAM mutations, and one of the three sets of allele-defining SNVs (wild-type, methylated or unmethylated) as well as unmatched sequences.

**Additional file 5: Figure S4.** Nested PCR from Bisulfite-Converted Pre-selection, Mock Selected, or 6-TG Selected Cell Genomic DNA for Illumina Sequencing. Bisulfite-converted genomic DNA was the template for the first round of PCR. In this round, both primers were inside the CRISPR cut sites in the genome. A unique molecular index (UMI) and a primer binding site were added in this round of PCR. The product of this PCR was used as the template for the second round PCR.

**Additional file 6: Figure S5.** Percentage of reads with an indel at positions along the PacBio sequenced region, broken down by selection status (pre-selection and mock selected vs. 6-TG selected) and allele methylation type (methylated vs. unmethylated alleles). Similar criteria for inclusion of reads as in Fig. 2a and Fig. 3a were used to generate this figure (perfect match on the allele-defining SNVs and the surrounding portion of exon 1). Red arrowheads indicate the CRISPR/Cas9 cut sites. The purple bar marks the region of exon 1 surrounding the allele-defining SNVs. The distribution of indels is highest at the CRISPR/Cas9 cut sites, but many reads have indels extending into the CpG island as well. In particular, 6-TG selected unmethylated alleles (lower left panel) have higher percentages of large indel events extending into the CpG island.

**Additional file 7: Figure S6.** UCSC genome browser view showing the region around the transcriptional start of *HPRT1*, CpG dinucleotides included in the CpG island amplicons, locations of SNVs introduced to create alleles and destroy PAM sites, and location of the *HPRT1* CpG island.

**Additional file 8: Table S2.** Sequences of Primers and Oligonucleotides for Cloning.

## Data Availability

The datasets generated and/or analyzed during the current study are available in the NCBI SRA repository https://www.ncbi.nlm.nih.gov/bioproject/PRJNA547358 [24].

## Abbreviations

CRISPR: Clustered regularly interspaced short palindromic repeats
DSB: Double-strand break
dCas9: Catalytically inactive Cas9 protein
NHEJ: Non-homologous end joining
6-TG: 6-Thioguanine
SNV: Single nucleotide variant
PAM: Protospacer-associated motif
ORF: Open reading frame
FACS: Fluorescence-activated cell sorting
Bp: Base pair
DMSO: Dimethyl sulfoxide
PCR: Polymerase chain reaction
CDS: CoDing Sequence
UTR: Untranslated region
PacBio: Pacific Biosciences
CCS: Circular Consensus Sequence
UMI: Unique molecular identifier

## References

[1] Sander JD, Keith JJ (2014). CRISPR-Cas systems for editing, regulating and targeting genomes. Nat Biotechnol.

[2] Tsai SQ, Zheng Z, Nguyen NT, Liebers M, Topkar VV, Thapar V (2015). GUIDE-seq enables genome-wide profiling of off-target cleavage by CRISPR-Cas nucleases. Nat Biotechnol.

[3] Geisinger JM, Turan S, Hernandez S, Spector LP, Calos MP (2016). In vivo blunt-end cloning through CRISPR/Cas9-facilitated non-homologous end-joining. Nucleic Acids Res.

[4] Schübeler D (2015). Function and information content of DNA methylation. Nature..

[5] Jones PA (2012). Functions of DNA methylation: islands, start sites, gene bodies and beyond. Nat Rev Genet.

[6] Bergman Y, Cedar H (2013). DNA methylation dynamics in health and disease. Nat Struct Mol Biol.

[7] Jin Z, Liu Y (2018). DNA methylation in human diseases. Genes Diseases.

[8] Lorincz MC, Schübeler D, Groudine M (2001). Methylation-mediated proviral silencing is associated with MeCP2 recruitment and localized histone H3 deacetylation. Mol Cell Biol.

[9] Lorincz MC, Schübeler D, Hutchinson SR, Dickerson DR, Groudine M (2002). DNA methylation density influences the stability of an epigenetic imprint and Dnmt3a/b-independent de novo methylation. Mol Cell Biol.

[10] Schübeler D, Lorincz MC, Cimbora DM, Telling A, Feng YQ, Bouhassira EE (2000). Genomic targeting of methylated DNA: influence of methylation on transcription, replication, chromatin structure, and histone acetylation. Mol Cell Biol.

[11] Liu XS, Wu H, Ji X, Stelzer Y, Wu X, Czauderna S (2016). Editing DNA methylation in the mammalian genome. Cell.

[12] Amabile A, Migliara A, Capasso P, Biffi M, Cittaro D, Naldini L (2016). Inheritable silencing of endogenous genes by hit-and-run targeted epigenetic editing. Cell.

[13] Vojta A, Dobrinić P, Tadić V, Bočkor L, Korać P, Julg B (2016). Repurposing the CRISPR-Cas9 system for targeted DNA methylation. Nucleic Acids Res.

[14] McDonald JI, Celik H, Rois LE, Fishberger G, Fowler T, Rees R (2016). Reprogrammable CRISPR/Cas9-based system for inducing site-specific DNA methylation. Biol Open.

[15] Stepper P, Kungulovski G, Jurkowska RZ, Chandra T, Krueger F, Reinhardt R (2017). Efficient targeted DNA methylation with chimeric dCas9-Dnmt3a-Dnmt3L methyltransferase. Nucleic Acids Res.

[16] Lei Y, Zhang X, Su J, Jeong M, Gundry MC, Huang Y-H (2017). Targeted DNA methylation in vivo using an engineered dCas9-MQ1 fusion protein. Nat Commun.

[17] Xiong T, Meister GE, Workman RE, Kato NC, Spellberg MJ, Turker F (2017). Targeted DNA methylation in human cells using engineered dCas9-methyltransferases. Sci Rep.

[18] Adli M (2018). The CRISPR tool kit for genome editing and beyond. Nat Commun.

[19] Gasperini M, Findlay GM, McKenna A, Milbank JH, Lee C, Zhang MD (2017). CRISPR/Cas9-mediated scanning for regulatory elements required for HPRT1 expression via thousands of large, programmed genomic deletions. Am J Hum Genet.

[20] Thongsroy J, Matangkasombut O, Thongnak A, Rattanatanyong P, Jirawatnotai S, Mutirangura A (2013). Replication-independent endogenous DNA double-strand breaks in Saccharomyces cerevisiae model. PLoS One.

[21] Kongruttanachok N, Phuangphairoj C, Thongnak A, Ponyeam W, Rattanatanyong P, Pornthanakasem W (2010). Replication independent DNA double-strand break retention may prevent genomic instability. Mol Cancer.

[22] Guo T, Feng Y-L, Xiao J-J, Liu Q, Sun X-N, Xiang J-F (2018). Harnessing accurate non-homologous end joining for efficient precise deletion in CRISPR/Cas9-mediated genome editing. Genome Biol.

[23] Jiang H, Lei R, Ding S-W, Zhu S (2014). Skewer: a fast and accurate adapter trimmer for next-generation sequencing paired-end reads. BMC Bioinformatics.

[24] Alexander J, Findlay FM, Kircher M, Shendure J. Concurrent genome and epigenome editing by CRISPR-mediated sequence replacement of the HPRT1 CpG island. NCBI SRA. PRJNA547358. https://www.ncbi.nlm.nih.gov/bioproject/PRJNA547358. 10.1186/s12915-019-0711-z PMC6862751 31739790

