# Supplementary figures and images for: Concurrent genome and epigenome editing by CRISPR-mediated sequence replacement
Source: BMC Biol. 2019 Nov 18;17:90. doi: 10.1186/s12915-019-0711-z (PMC6862751; doi:10.1186/s12915-019-0711-z)

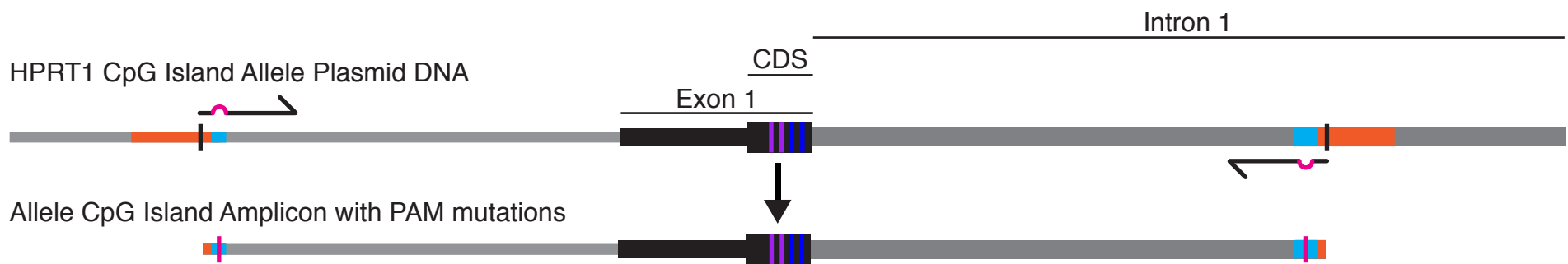

Note: Graphics are not to scale.

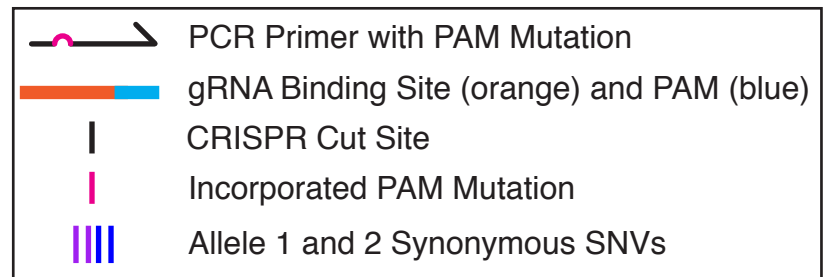

Supplement: Supplementary file 1 — Additional file 1: Figure S1. PCR for generation of CpG Island Allele Amplicon DNA with PAM mutations. Cloned HPRT1 CpG island plasmid DNA was used as a template for PCR amplification. PAM sites corresponding to the guide RNA target sites are at the ends within the CpG island amplicon. Primer sequences included a mismatch near the 5′ end resulting in incorporation of a mutation in the PAM sequences at the ends of the CpG island allele amplicons. [file 12915_2019_711_MOESM1_ESM.pdf]

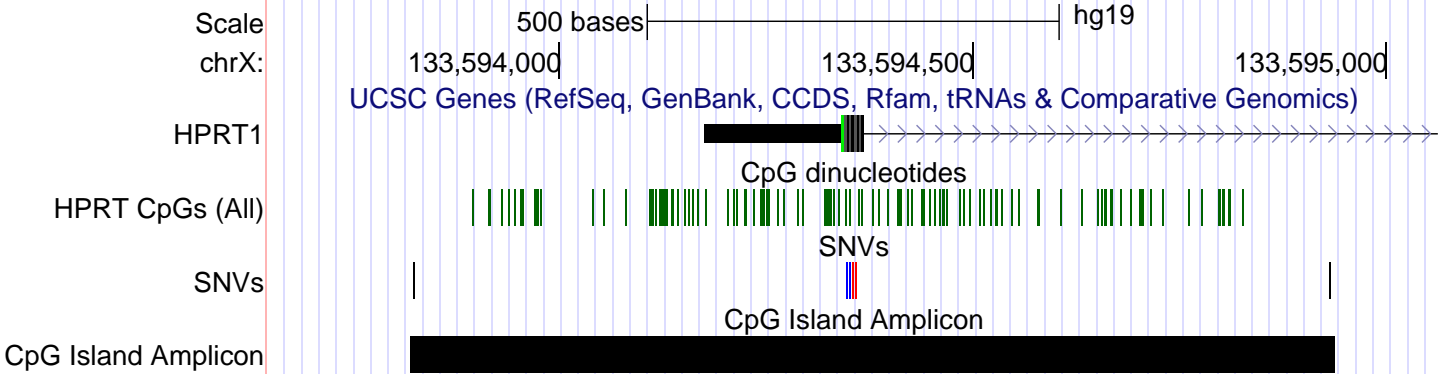

Supplement: Supplementary file 7 — Additional file 7: Figure S6. UCSC genome browser view showing the region around the transcriptional start of HPRT1, CpG dinucleotides included in the CpG island amplicons, locations of SNVs introduced to create alleles and destroy PAM sites, and location of the HPRT1 CpG island. [file 12915_2019_711_MOESM7_ESM.pdf]
