# Supplementary material for: Concurrent genome and epigenome editing by CRISPR-mediated sequence replacement
Source: BMC Biol. 2019 Nov 18;17:90. doi: 10.1186/s12915-019-0711-z (PMC6862751; doi:10.1186/s12915-019-0711-z)
Supplement: Supplementary file 2 — Additional file 2: Figure S2. Nested PCR from Pre-selection, Mock Selected, or 6-TG Selected Cell Genomic DNA for Illumina Sequencing. Genomic DNA was the template for the first round of PCR. In this round, one primer was outside the CRISPR cut sites in the genome, while the other primer was within the cut sites in the HPRT1 CpG island. The product of the PCR was used as the template for the second round PCR. The second round PCR amplified a 44-bp region including the allele-defining SNVs. [file 12915_2019_711_MOESM2_ESM.pdf]

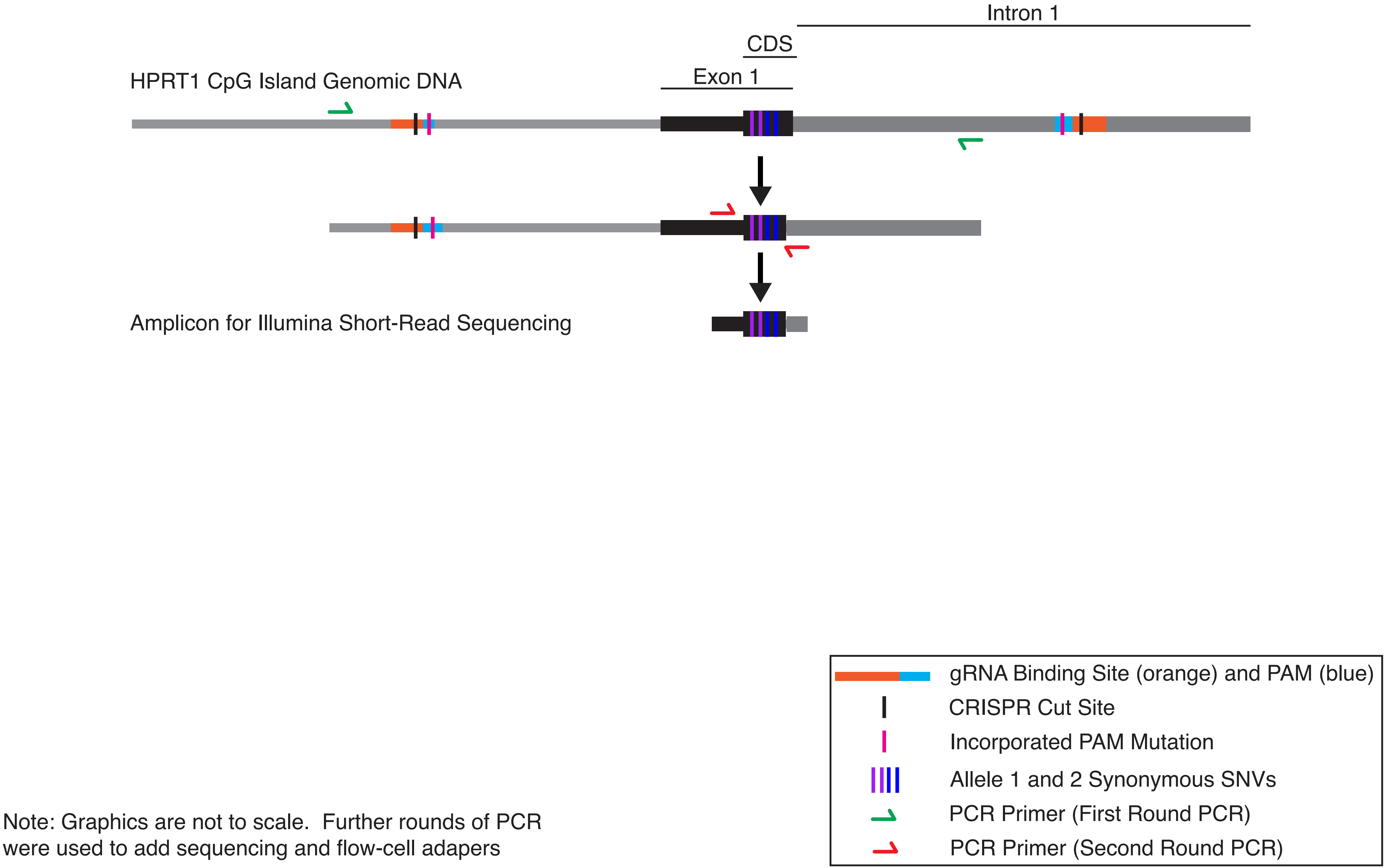
Intron 1
CDS
Exon 1
HPRT1 CpG Island Genomic DNA
Amplicon for Illumina Short-Read Sequencing
gRNA Binding Site (orange) and PAM (blue)
CRISPR Cut Site
Incorporated PAM Mutation
Allele 1 and 2 Synonymous SNVs
PCR Primer (First Round PCR)
PCR Primer (Second Round PCR)
Note: Graphics are not to scale. Further rounds of PCR
were used to add sequencing and flow-cell adapers
