# Supplementary material for: Concurrent genome and epigenome editing by CRISPR-mediated sequence replacement
Source: BMC Biol. 2019 Nov 18;17:90. doi: 10.1186/s12915-019-0711-z (PMC6862751; doi:10.1186/s12915-019-0711-z)
Supplement: Supplementary file 3 — Additional file 3: Figure S3. PCR from Pre-selection, Mock Selected, or 6-TG Selected Cell Genomic DNA for Pacific Biosciences Sequencing. Genomic DNA was the template for the first round of PCR. In this round, both primers were outside the CRISPR cut sites in the genome. A unique molecular index (UMI) and a primer binding site were added in this round of PCR. The product of this PCR was used as the template for the second round PCR. [file 12915_2019_711_MOESM3_ESM.pdf]

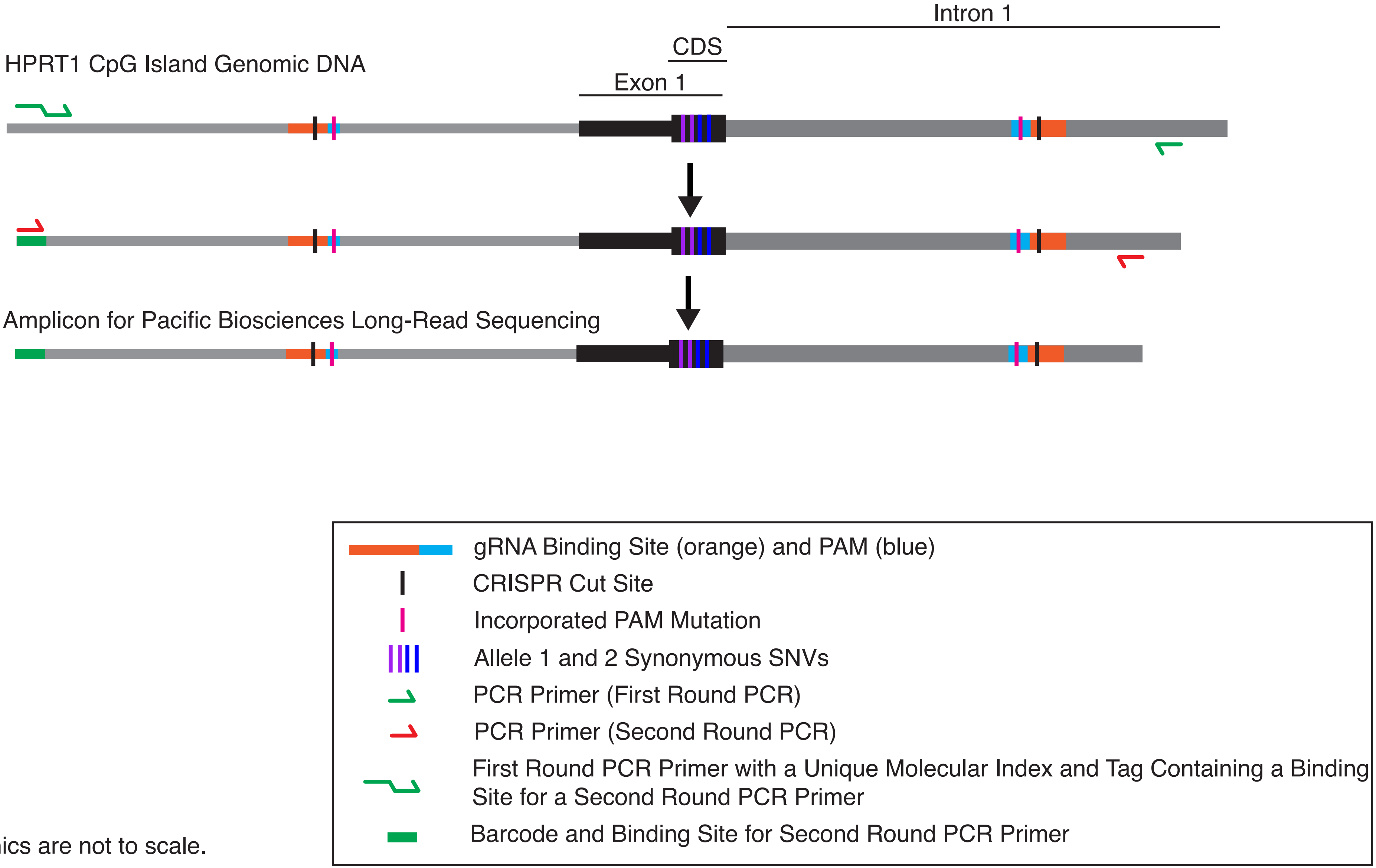

Note: Graphics are not to scale.
