# Supplementary material for: Concurrent genome and epigenome editing by CRISPR-mediated sequence replacement
Source: BMC Biol. 2019 Nov 18;17:90. doi: 10.1186/s12915-019-0711-z (PMC6862751; doi:10.1186/s12915-019-0711-z)
Supplement: Supplementary file 5 — Additional file 5: Figure S4. Nested PCR from Bisulfite-Converted Pre-selection, Mock Selected, or 6-TG Selected Cell Genomic DNA for Illumina Sequencing. Bisulfite-converted genomic DNA was the template for the first round of PCR. In this round, both primers were inside the CRISPR cut sites in the genome. A unique molecular index (UMI) and a primer binding site were added in this round of PCR. The product of this PCR was used as the template for the second round PCR. [file 12915_2019_711_MOESM5_ESM.pdf]

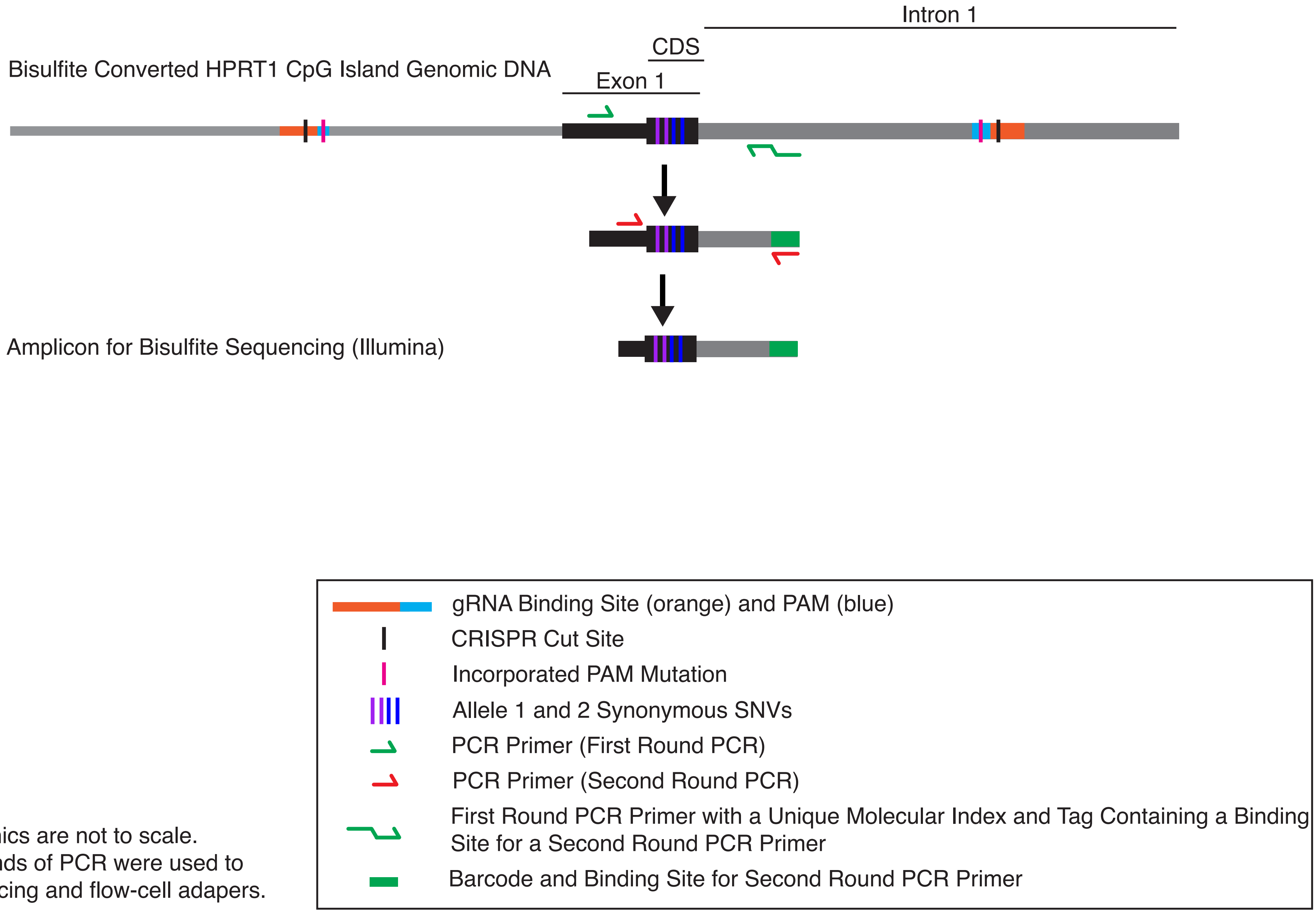

Note: Graphics are not to scale. Further rounds of PCR were used to add sequencing and flow-cell adapers.
