# Supplementary material for: Concurrent genome and epigenome editing by CRISPR-mediated sequence replacement
Source: BMC Biol. 2019 Nov 18;17:90. doi: 10.1186/s12915-019-0711-z (PMC6862751; doi:10.1186/s12915-019-0711-z)
Supplement: Supplementary file 6 — Additional file 6: Figure S5. Percentage of reads with an indel at positions along the PacBio sequenced region, broken down by selection status (pre-selection and mock selected vs. 6-TG selected) and allele methylation type (methylated vs. unmethylated alleles). Similar criteria for inclusion of reads as in Fig. 2a and Fig. 3a were used to generate this figure (perfect match on the allele-defining SNVs and the surrounding portion of exon 1). Red arrowheads indicate the CRISPR/Cas9 cut sites. The purple bar marks the region of exon 1 surrounding the allele-defining SNVs. The distribution of indels is highest at the CRISPR/Cas9 cut sites, but many reads have indels extending into the CpG island as well. In particular, 6-TG selected unmethylated alleles (lower left panel) have higher percentages of large indel events extending into the CpG island. [file 12915_2019_711_MOESM6_ESM.pdf]

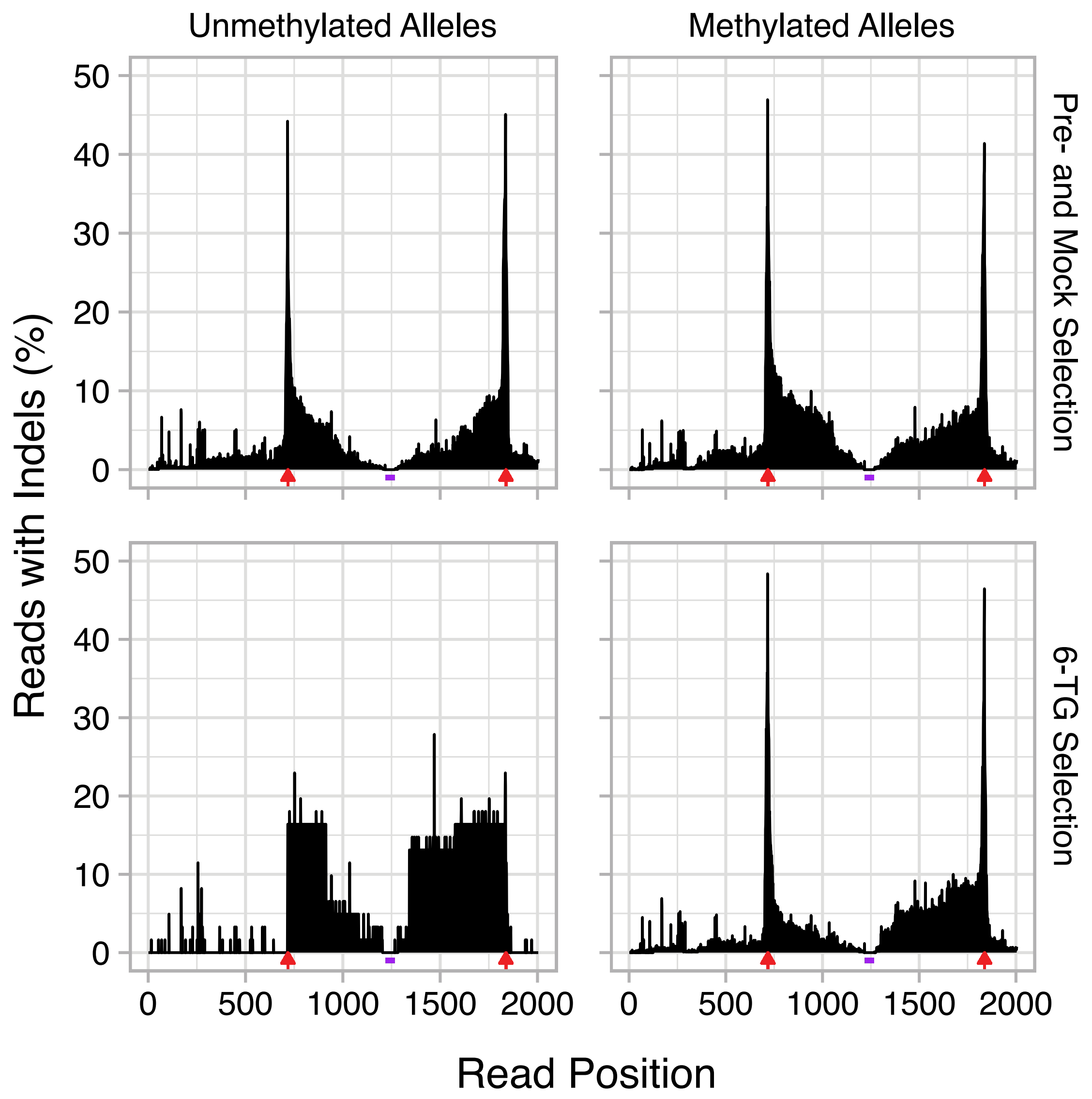
Unmethylated Alleles
Methylated Alleles
Pre- and Mock Selection
6-TG Selection
Reads with Indels (%)
Read Position
0
10
20
30
40
50
0
500
1000
1500
2000
