# Supplementary material for: Concurrent genome and epigenome editing by CRISPR-mediated sequence replacement
Source: BMC Biol. 2019 Nov 18;17:90. doi: 10.1186/s12915-019-0711-z (PMC6862751; doi:10.1186/s12915-019-0711-z)
Supplement: Supplementary file 8 — Additional file 8: Table S2. Sequences of Primers and Oligonucleotides for Cloning. [file 12915_2019_711_MOESM8_ESM.docx]

**HPRT1 CpG island region genomic amplification:**

1. (JA-Me-HPRT-gen-1-f): CGG TAC CCG GGG ATC-gtgaggcaaaaatagaggctcagagt

2. (JA-ME-HPRT-gen-1-R): CGA CTC TAG AGG ATC GCA GCT TGG CCG GTT CAA CAAA

**Synonymous SNVs:**

Allele 1 (SNVs at 133594350 and 133594359)

3. Forward (JA-Me-HPRT-Mut-6-f): T-CGCAGCCC-A-GGCGT-CGTGGTGAGCAGCTCGGC

4. Reverse (JA-Me-HPRT-Mut-6-r): ACG CCT GGG CTG CGA- GTCGCCATAACGGAGCCG

Allele 2 (SNVs at 133594353 and 133594356)

5. Forward (JA-Me-HPRT-Mut-7-f): CCG-G-AG-T-CCTGGCGT-CGTGGTGAGCAGCTCGGC

6. Reverse (JA-Me-HPRT-Mut-7-r): ACG CCA GGA CTC CGG-GTCGCCATAACGGAGCCG

**Oligonucleotides for cloning guide RNAs:**

chrX:133593802-133593821

7. (JA-Me-HPRT-sgRNA5’-1-f): CACC-GCAACTGTTACAACCAGTTAA

8. (JA-Me-HPRT-sgRNA5’-1-r): aaac-TTAACTGGTTGTAACAGTTG-C

chrX:133594936-133594955

9. (JA-Me-HPRT-sgRNA3’-1-f): CACCGcttcgtgtgtcaaatacgca

10. (JA-Me-HPRT-sgRNA3’-1-r): AAAC-tgcgtatttgacacacgaag-C

**NHEJ Template Amplicon:**

11. (JA-ME-HPRT-Nokin-1*-f):T*A*A*GG-C-TTTGGGGAAGCACTG

12. (JA-ME-HPRT-Nokin-1*-r): g*c*a*tg-c-ttaccgctaccagag

* = phosphorothioate linkage

**Illumina Sequencing:**

Round 1

13. (JA-ME-HPRT-onDNA-1-f): GTGAGGCAAAAATAGAGGCTCAGAGT

14. (JA-ME-HPRT-onDNA-2-r): CAGTGTGTGCAAAACTAAAGGCA

Round 2

15. (JA-ME-HPRT-cDNA-1-f): CTAAATGGCTGTGAGAGAGCTCAG-TTCCTCCTCCTGAGCAGTCAGC

16. (JA-ME-HPRT-DNA-1-r): ACTTTATCAATCTCGCTCCAAACC-CGAGCTGCTCACCACGA

Round 3 Reverse

17. (jkA0142_ampRbnew1_codv): CAAGCAGAAGACGGCATACGAGAT AAGCGTTCA gaccgtcggc ACTTTATCAATCTCGCTCCAAACC

18. (jkA0143_ampRbnew2_codv): CAAGCAGAAGACGGCATACGAGAT CGCAAGCGT gaccgtcggc ACTTTATCAATCTCGCTCCAAACC

19. (jkA0144_ampRbnew3_codv): CAAGCAGAAGACGGCATACGAGAT GCAGCGCGA gaccgtcggc ACTTTATCAATCTCGCTCCAAACC

20. (jkA0145_ampRbnew4_codv): CAAGCAGAAGACGGCATACGAGAT CGCGCAGCT gaccgtcggc ACTTTATCAATCTCGCTCCAAACC

21. (jkA0146_ampRbnew5_codv): CAAGCAGAAGACGGCATACGAGAT TCAAGCGCA gaccgtcggc ACTTTATCAATCTCGCTCCAAACC

22. (jkA0147_ampRbnew6_codv): CAAGCAGAAGACGGCATACGAGAT CAGTCGCAG gaccgtcggc ACTTTATCAATCTCGCTCCAAACC

23. (jkA0148_ampRbnew7_codv): CAAGCAGAAGACGGCATACGAGAT GCGTCAGTT gaccgtcggc ACTTTATCAATCTCGCTCCAAACC

24. (jkA0149_ampRbnew8_codv): CAAGCAGAAGACGGCATACGAGAT AGTCGCGCA gaccgtcggc ACTTTATCAATCTCGCTCCAAACC

Round 3 Forward

25. (P5_pu1Lfwd_fwd_GF_P5barc_03): AATGATACGGCGACCACCGAGATCTACAC AAATCTGCGT acgtaggc CTAAATGGCTGTGAGAGAGCTCAG

26. (P5_pu1Lfwd_fwd_GF_P5barc_04): AATGATACGGCGACCACCGAGATCTACAC

ATTTAGTACG acgtaggc CTAAATGGCTGTGAGAGAGCTCAG

27. (P5_pu1Lfwd_fwd_GF_P5barc_05): AATGATACGGCGACCACCGAGATCTACAC

CCACACAAGC acgtaggc CTAAATGGCTGTGAGAGAGCTCAG

**Pacific Biosciences RSII Sequencing:**

Round 1

28. (JA-MeHPRT-InDel-Out1-f): CTAAATGGCTGTGAGAGAGCTCAG-NNNNN NNNNN-AATGGTGTTGCTGGAGCAACTGTT

29. (JA-MeHPRT-InDel-Out1-r): GCAGGCTAAAGCATATTTAACTGGC

Round 2 and 3

30. (JA-MeHPRT-InDel-In1-f): GGTGGT-GAATTC-CTAAATGGCTGTGAGAGAGCTCAG

31. (JA-MeHPRT-InDel-In1-r): GGTGGT-CCTGCAGG- GTGGAGCTAAGATACCAGAGGCTG

**Pacific Biosciences Sequel Sequencing:**

Round 1 Forward

32. (JA-MeHPRT-142-Out1-f): CTAAATGGCTGTGAGAGAGCTCAG-TGAACGCTT-NNNNNNNNNN-AATGGTGTTGCTGGAGCAACTGTT

33. (JA-MeHPRT-143-Out1-f): CTAAATGGCTGTGAGAGAGCTCAG-ACGCTTGCG-NNNNNNNNNN-AATGGTGTTGCTGGAGCAACTGTT

34. (JA-MeHPRT-144-Out1-f): CTAAATGGCTGTGAGAGAGCTCAG-TCGCGCTGC-NNNNNNNNNN-AATGGTGTTGCTGGAGCAACTGTT

35. (JA-MeHPRT-145-Out1-f): CTAAATGGCTGTGAGAGAGCTCAG-AGCTGCGCG-NNNNNNNNNN-AATGGTGTTGCTGGAGCAACTGTT

36. (JA-MeHPRT-146-Out1-f): CTAAATGGCTGTGAGAGAGCTCAG-TGCGCTTGA-NNNNNNNNNN-AATGGTGTTGCTGGAGCAACTGTT

37. (JA-MeHPRT-147-Out1-f): CTAAATGGCTGTGAGAGAGCTCAG-CTGCGACTG-NNNNN NNNNN-AATGGTGTTGCTGGAGCAACTGTT

38. (JA-MeHPRT-InDel_148-Out1-f): CTAAATGGCTGTGAGAGAGCTCAG-AACTGACGC-NNNNNNNNNN-AATGGTGTTGCTGGAGCAACTGTT

39. (JA-MeHPRT-InDel_149-Out1-f): CTAAATGGCTGTGAGAGAGCTCAG-TGCGCGACT-NNNNNNNNNN-AATGGTGTTGCTGGAGCAACTGTT

40. (JA-MeHPRT-InDel_151-Out1-f): CTAAATGGCTGTGAGAGAGCTCAG-ACATCGTAG-NNNNNNNNNN-AATGGTGTTGCTGGAGCAACTGTT

41. (JA-MeHPRT-InDel_152-Out1-f): CTAAATGGCTGTGAGAGAGCTCAG-TAGCAGGAT-NNNNN NNNNN-AATGGTGTTGCTGGAGCAACTGTT

42. (JA-MeHPRT-InDel_153-Out1-f): CTAAATGGCTGTGAGAGAGCTCAG-ATCGTAGCC-NNNNNNNNNN-AATGGTGTTGCTGGAGCAACTGTT

43. (JA-MeHPRT-InDel_154-Out1-f): CTAAATGGCTGTGAGAGAGCTCAG-TCTATTCAC-NNNNNNNNNN-AATGGTGTTGCTGGAGCAACTGTT

44. (JA-MeHPRT-InDel_155-Out1-f): CTAAATGGCTGTGAGAGAGCTCAG-CGTCTCCTA-NNNNNNNNNN-AATGGTGTTGCTGGAGCAACTGTT

45. (JA-MeHPRT-InDel_156-Out1-f): CTAAATGGCTGTGAGAGAGCTCAG-AGGTGATTC-NNNNNNNNNN-AATGGTGTTGCTGGAGCAACTGTT

Round 1 Reverse

29. (JA-MeHPRT-InDel-Out1-r): GCAGGCTAAAGCATATTTAACTGGC

Round 2 and 3

30. (JA-MeHPRT-InDel-In1-f): GGTGGT-GAATTC- CTAAATGGCTGTGAGAGAGCTCAG

31. (JA-MeHPRT-InDel-In1-r) : GGTGGT-CCTGCAGG- GTGGAGCTAAGATACCAGAGGCTG

**Bisulfite Sequencing:**

Round 1

46. (JA-ME-HPRT-BCOutIll-2-f):CTAAATGGCTGTGAGAGAGCTCAG-NNNNN NNNNN-AGGAGGGAtttAttttAAAttt

47. (JA-ME-HPRT-BCOutIll-2-r): CCTCCTCCTCTaCTCc

Round 2

48. (JA-ME-HPRT-BCInIll-2-f): CTAAATGGCTGTGAGAGAGCTCAG

49. (JA-ME-HPRT-BCInIll-2-r): ACTTTATCAATCTCGCTCCAAACC-aCTTCCTCCTCCTaAaCAaTCAaCC

Round 3 Reverse

17. (jkA0142_ampRbnew1_codv): CAAGCAGAAGACGGCATACGAGAT AAGCGTTCA gaccgtcggc ACTTTATCAATCTCGCTCCAAACC

18. (jkA0143_ampRbnew2_codv): CAAGCAGAAGACGGCATACGAGAT CGCAAGCGT gaccgtcggc ACTTTATCAATCTCGCTCCAAACC

19. (jkA0144_ampRbnew3_codv): CAAGCAGAAGACGGCATACGAGAT GCAGCGCGA gaccgtcggc ACTTTATCAATCTCGCTCCAAACC

20. (jkA0145_ampRbnew4_codv): CAAGCAGAAGACGGCATACGAGAT CGCGCAGCT gaccgtcggc ACTTTATCAATCTCGCTCCAAACC

21. (jkA0146_ampRbnew5_codv): CAAGCAGAAGACGGCATACGAGAT TCAAGCGCA gaccgtcggc ACTTTATCAATCTCGCTCCAAACC

22. (jkA0147_ampRbnew6_codv): CAAGCAGAAGACGGCATACGAGAT CAGTCGCAG gaccgtcggc ACTTTATCAATCTCGCTCCAAACC

23. (jkA0148_ampRbnew7_codv): CAAGCAGAAGACGGCATACGAGAT GCGTCAGTT gaccgtcggc ACTTTATCAATCTCGCTCCAAACC

24. (jkA0149_ampRbnew8_codv): CAAGCAGAAGACGGCATACGAGAT AGTCGCGCA gaccgtcggc ACTTTATCAATCTCGCTCCAAACC

Round 3 Forward

50. (P5_pu1Lfwd_fwd_GF_P5barc_01): AATGATACGGCGACCACCGAGATCTACAC

TAAATGCTCC acgtaggc CTAAATGGCTGTGAGAGAGCTCAG

**Illumina Sequencing Primers:**

51. (PU1_SEQ_F_R1): acgtaggcCTAAATGGCTGTGAGAGAGCTCAG

52. (PU1_SEQ_R_R2): gaccgtcggcACTTTATCAATCTCGCTCCAAACC

53. (PU1_seqIx1): GGTTTGGAGCGAGATTGATAAAGTgccgacggtc
